# Transcatheter Tricuspid Valve Embolization: Revealing Critical Limitations of Contemporary Valve Design

**DOI:** 10.1016/j.jaccas.2026.109402

**Published:** 2026-07-17

**Authors:** Seyed Hossein Aalaei-Andabili, Douglas Anderson, Sara G. Kwiatkowski, Diljon S. Chahal

**Affiliations:** a Division of Cardiology, Department of Medicine, University of Maryland Medical Center, Baltimore, Maryland, USA; b Division of Cardiac Surgery, Department of Surgery, University of Maryland Medical Center, Baltimore, Maryland, USA

**Keywords:** embolization, Evoque, transcatheter tricuspid valve, valve design

## Abstract

**Background:**

Edwards Lifesciences' Evoque valve system is the only approved transcatheter system for tricuspid valve replacement (TTVR). Two major studies have shown remarkable improvements in quality of life after the Evoque valve replacement, and a 2-year follow-up suggested a mortality benefit.

**Case Summary:**

A 64-year-old woman with severe symptomatic tricuspid valve regurgitation underwent implantation of an Evoque valve. Following the procedure, she developed frequent cardiac arrhythmia. Subsequent transthoracic and transesophageal echocardiograms demonstrated valve embolization. Intraoperative observation further revealed that a large ventricular septal defect was created by the displaced valve.

**Discussion:**

TTVR carries significant risks; valve migration can result in life-threatening complications, requiring immediate surgical evaluation. This report is the first to document a life-threatening valve embolization, indicating that the valve design remains suboptimal.

**Take-Home Messages:**

The current TTVR system design is inadequate. Valve migration is a potentially life-threatening complication that exposes fundamental flaws in the existing device design.

## Visual Summary

**Figure:**
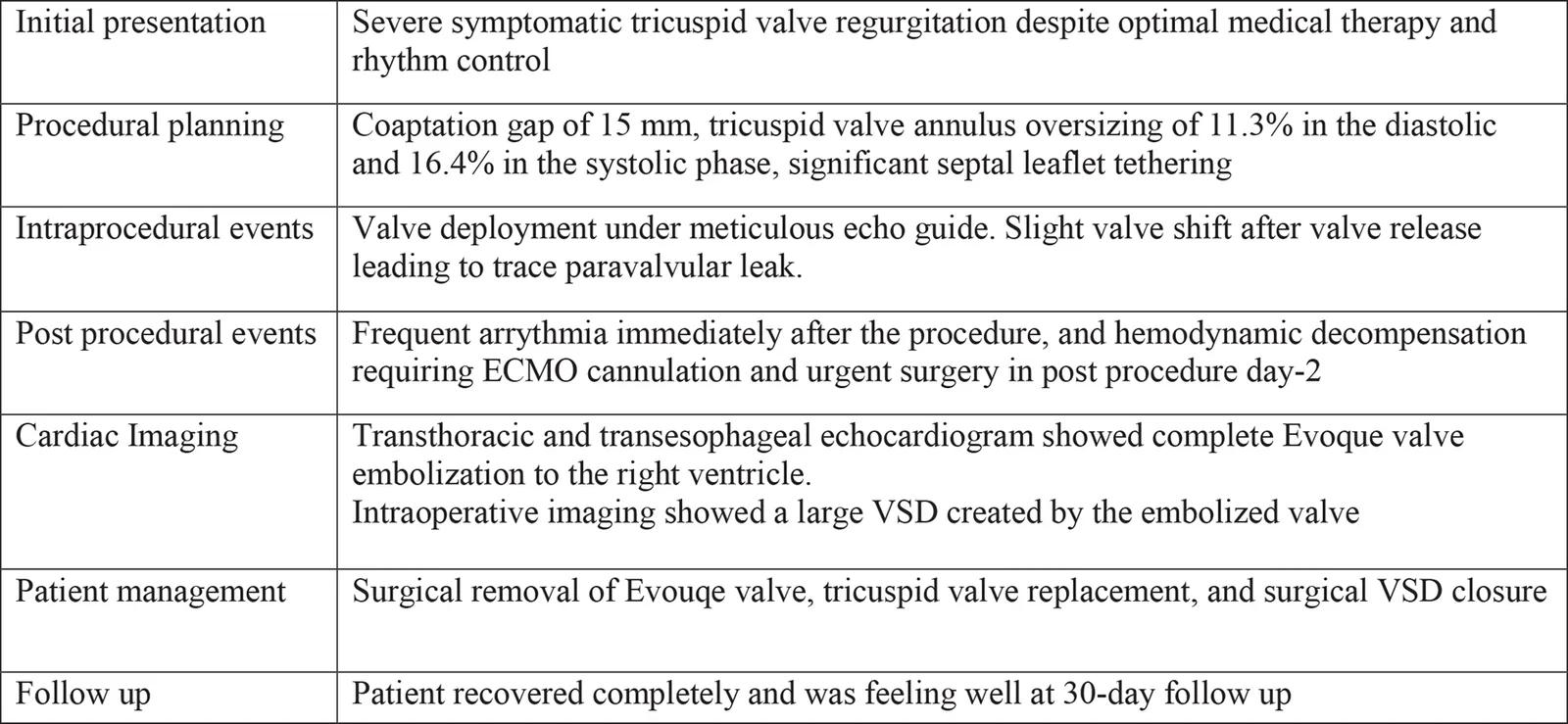

## History of Presentation

A 64-year-old woman with severe symptomatic tricuspid valve regurgitation underwent transcatheter tricuspid valve replacement (TTVR) after optimizing medical therapy for cardiac risk factors and rhythm control. Before the TTVR, the patient underwent right heart catheterization, coronary angiography, pulmonary embolism, and cardiac amyloid evaluation, which did not reveal any significant abnormality. Preprocedural echocardiogram showed normal left ventricular function with an ejection fraction of 55% to 60%, mildly dilated right ventricle (RV) with mildly decreased right ventricular systolic function, and severe tricuspid valve regurgitation.

**Take-Home Message**

• Despite advances in TTVR technology, current valve designs remain suboptimal.
• Continued innovation in tricuspid valve interventions is necessary to reduce the valve-related trauma, enhance device performance, and improve overall procedural safety.

The patient was evaluated for transcatheter tricuspid edge-to-edge repair, but she was not a candidate because of large coaptation gaps with more than 15 mm. Therefore, TTVR was chosen as the preferable treatment approach. Computed tomography (CT) scan of the heart showed appropriate anatomy for TTVR with tricuspid valve annulus oversizing of 11.3% in the diastolic phase and 16.4% in the systolic phase. Significant septal leaflet tethering was noted on the CT evaluation.

**Figure 1:**
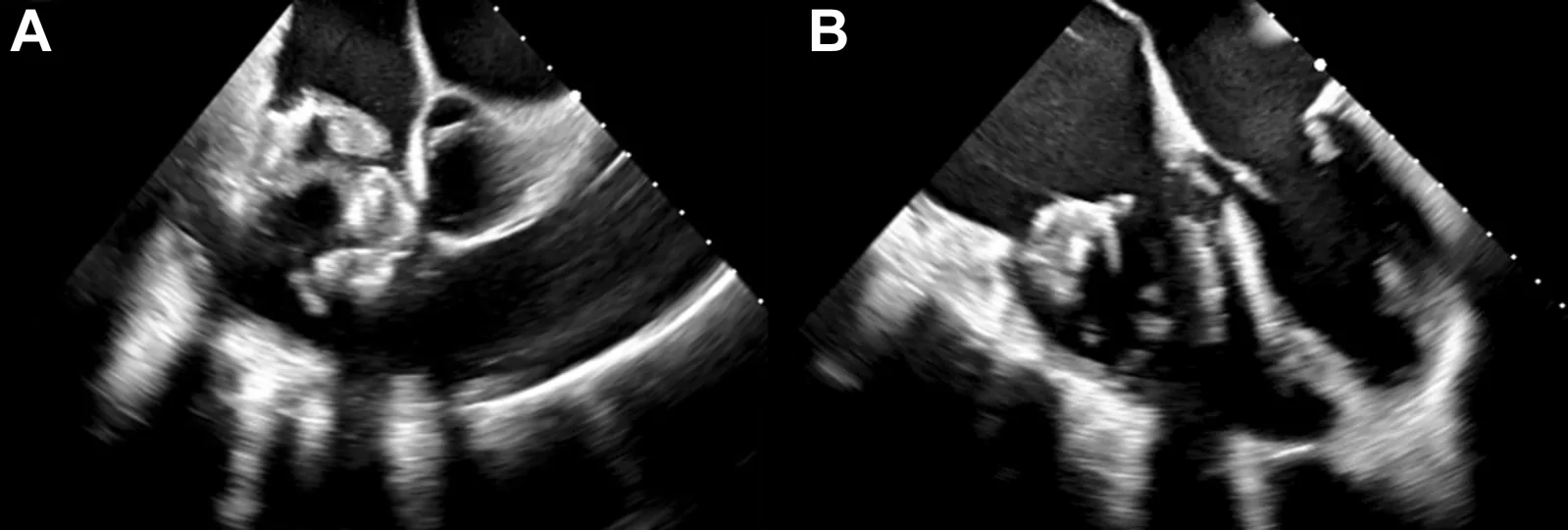
Transesophageal Echocardiography Shows Valve Embolization in 2 Views (A and B) Transesophageal echocardiography shows valve embolization in 2 views.

Intraprocedurally, valve placement was confirmed with multiple views and evaluations to ensure all leaflets were captured. As soon as the valve was fully released, there was a slight shift in the valve position, resulting in a trace paravalvular leak. The valve was reevaluated, and location and function remained appropriate before leaving the procedure room. Following transfer to the postprocedural area for recovery, the patient developed frequent premature ventricular beats but remained stable and otherwise asymptomatic. Later, she developed nonsustained ventricular tachycardia that required medical therapy with antiarrhythmic medications. On postoperative day 1, the echocardiogram showed embolization of the Evoque valve facing the interventricular septum, with a mild-to-moderate paravalvular leak. Transesophageal echocardiogram confirmed embolization of the valve to the right ventricle (Figures 1A and 1B).

## Past Medical History

The patient had past medical history of hypertension, atrial fibrillation, obstructive sleep apnea, diabetes mellitus, and anemia.

## Differential Diagnosis

The clear reason for valve embolization remains uncertain in this case. We explored the possibility of 1) not capturing all leaflets in the bioprosthetic valve during the release, 2) having part of the chordae within the Evoque valve structure, 3) or a highly oversized valve and further expansion after the release, as reasons for the valve embolization.

## Investigations

We observed a few entrapped tricuspid valve chordae in the Evoque valve intraoperatively, but this likely occurred after the embolization, as the valve was mobile with every heartbeat. The retrospective review of all intraoperative images revealed no chordae capture in the valve. Given all the assessments, we suspect that additional oversizing and subsequent expansion of the valve after release led to the valve tilt and eventually embolization, as the patient was better optimized in terms of volume before TTVR implantation vs the time CT scan for the of Evoque valve evaluation.

## Patient Management

**Figure 2:**
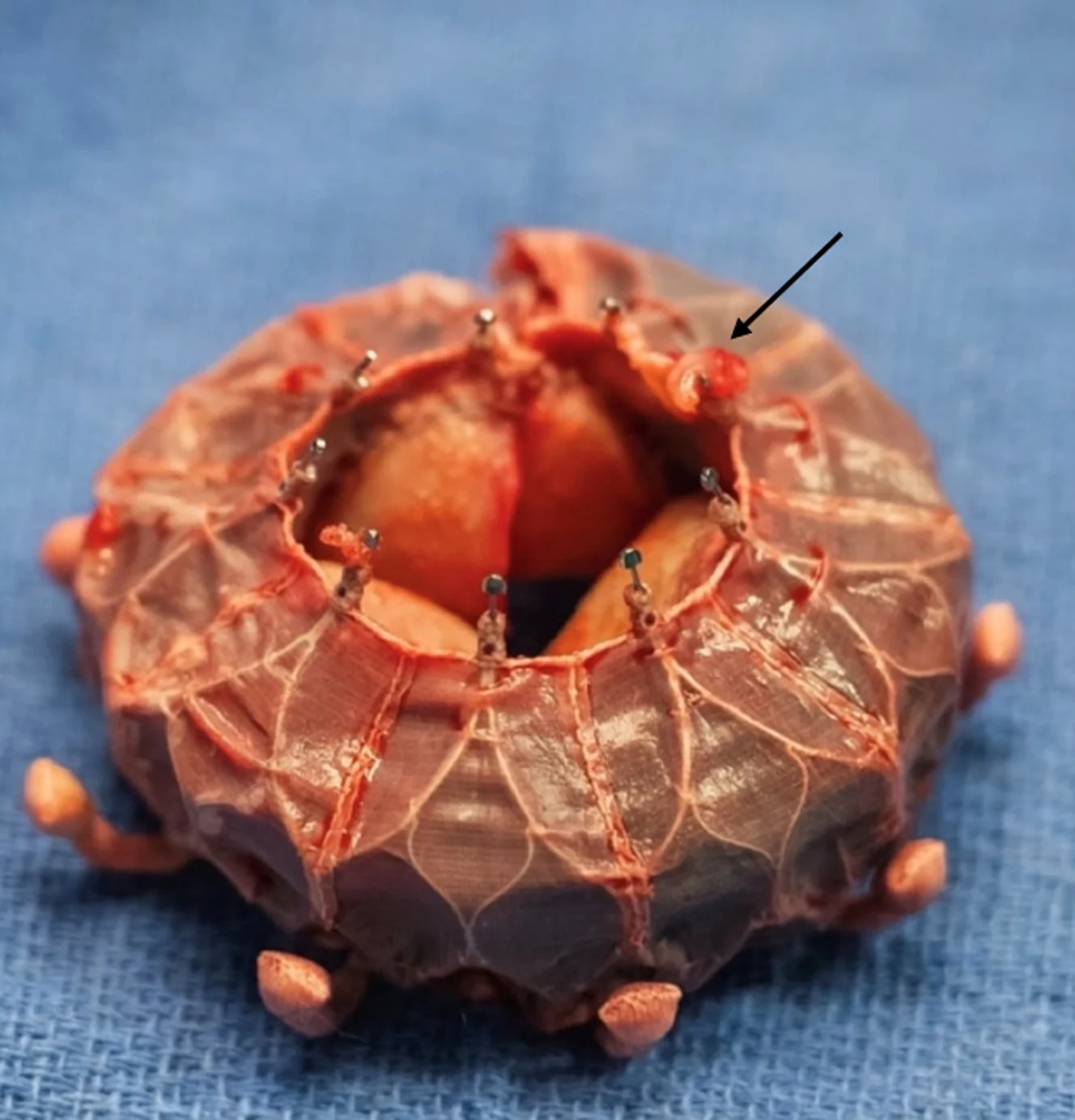
Interventricular Septal Muscle Mass Attached to the Valve Frame (Arrow)

**Figure 3:**
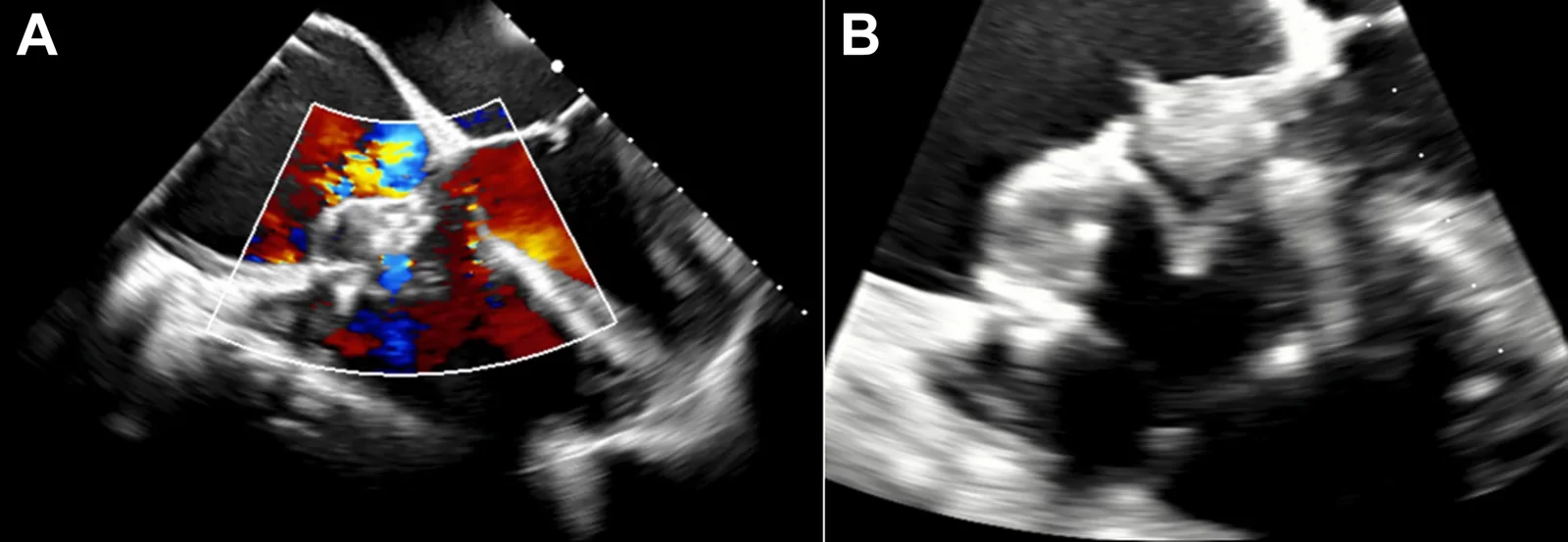
Transesophageal Echocardiography Shows Ventricular Septal Defect Created by the Embolized Evoque Valve (A and B) Ventricular septal defect created by the embolized Evoque valve.

**Figure 4:**
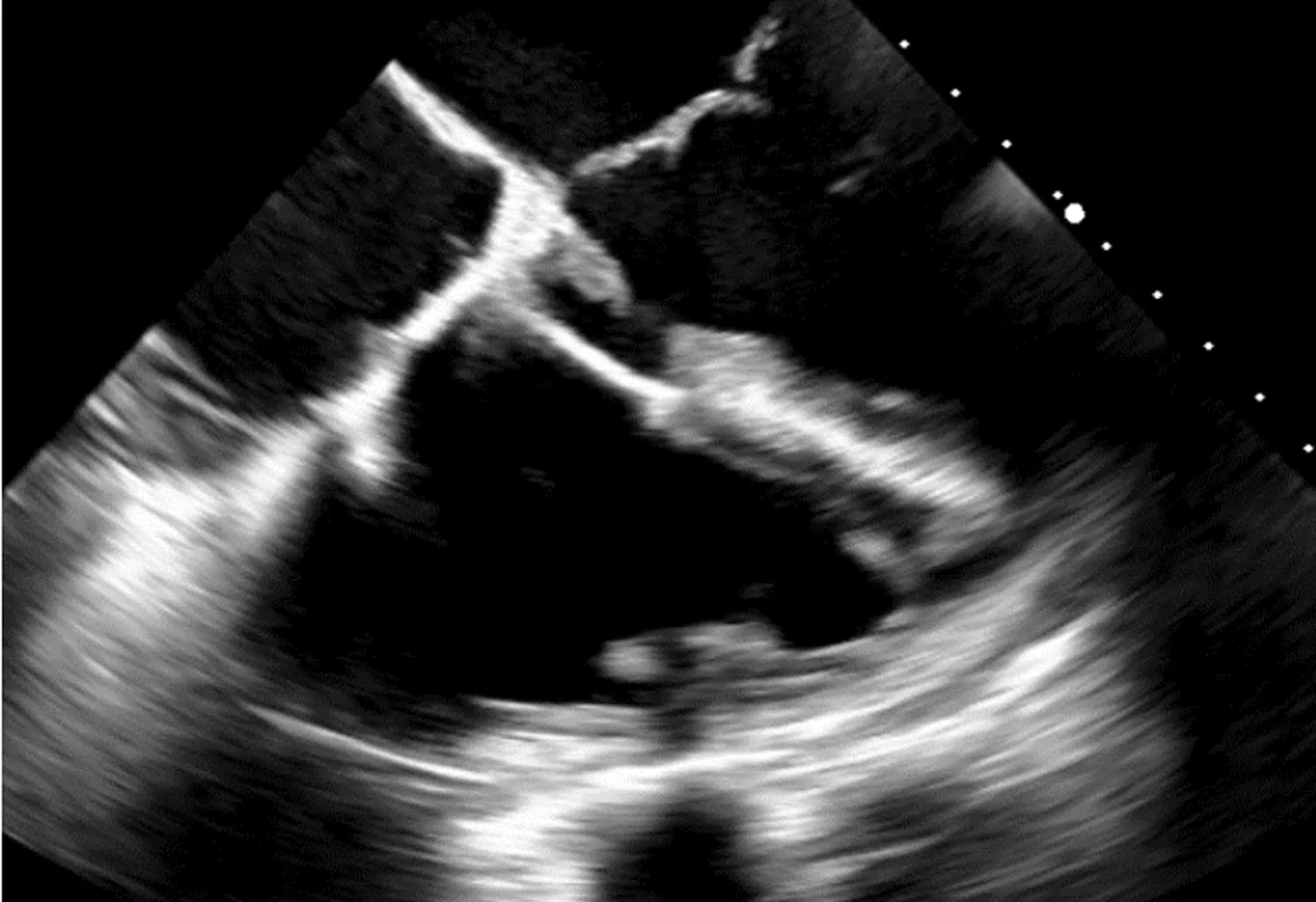
Surgically Repaired Ventricular Septal Defect

Following discussion with the patient and the interventional cardiology and cardiac surgery teams, the patient was found to be a surgical candidate for bioprosthetic valve extraction and native valve repair. Unfortunately, on postprocedure day 2, she suddenly became unresponsive, hypotensive, diaphoretic, and had cold extremities, raising concern for RV-related hemodynamic decompensation. At this point, immediate bedside extracorporeal membrane oxygenation cannulation was performed, and the patient was taken to the operation room emergently. Unexpectedly, a muscle mass was found on the valve (Figure 2) and a large new ventricular septal defect (VSD) was identified (Figures 3A and 3B). The tricuspid valve was replaced, and the VSD was repaired with the help of a congenital cardiac surgeon (Figure 4).

## Outcome and Follow-Up

The patient recovered well from the surgery and was discharged. At 30-day follow-up, the patient was doing great and echocardiogram showed normal biventricular function with no residual tricuspid regurgitation or VSD.

## Discussion

The Evoque valve system (Edwards Lifesciences) is the only TTVR system approved for commercial use by the U.S. Food and Drug Administration (FDA) since February 2024.^1^ Two major trials have shown quality-of-life and symptoms improvement after valve replacement with no mortality benefit.^2^ However, 2-year follow-up of TRISCEND II (Edwards EVOQUE Transcatheter Tricuspid Valve Replacement: Pivotal Clinical Investigation of Safety and Clinical Efficacy Using a Novel Device) trial shows all-cause mortality benefit after stratification of the control group based on crossover status.^3^ We are all still learning about valve replacement technical aspects and potential complications related to the Evoque valve. Bradycardia and high-degree atrioventricular node block were reported as the most common complications of the Evoque valve implantation followed by the valve migration and embolization.^4^ Although valve migration and embolization have been reported before, this is the first case of a life-threatening complication and Evoque valve–related VSD formation secondary to device embolization, which may help explain previously observed migration-related mortality and could be alarming for the current valve design.

From 158 adverse events submitted to the FDA, valve migration and embolization was reported in 20.9% of patients after Evoque valve implantation. More than 93% of these events occurred within 48 hours of the procedure.^5^ In the MAUDE (Manufacturer and User Facility Device Experience) database analysis by Lupu et al,^5^ there were 6 deaths of 33 (18.2%) patients with valve migration or embolization. However, the etiology and mechanism of mortality in these patients were not reported. Importantly, during intraoperative examination, the edges of the self-expanding nitinol frame were not atraumatic, and mechanical damage with each cardiac cycle by the valve nitinol frame led to traumatic muscular VSD formation.

Interestingly, we noticed intraoperatively that the valve itself was mostly attenuating the VSD flow when it was small, and the continued trauma to interventricular septum led to a larger VSD and hemodynamic decompensation on the day of surgery.

We were fortunate that the valve faced toward the septum rather than the RV lateral wall, which could have led to a life-threatening hemodynamically significant tamponade. These all suggest that the valve design for the transcatheter tricuspid valve is far from ideal, and further improvement is critical to make this procedure safer. Worth mentioning that the tricuspid valve has a unique anatomy with a mostly D-shaped annulus, and a single circular bioprosthetic valve might not fit everyone well, leading to improper anchoring of the Evoque valve and increasing risk of valve migration. The robust anchoring mechanism may play as a double-edged sword, enhancing device stability while increasing the risk of septal injury. Although VSD secondary to valve embolization might not occur frequently, making the valve design safer is necessary, as patients should not be at risk of a life-threatening complication for a procedure that has mostly shown quality-of-life improvement with possible survival benefit.

From a procedural standpoint, precise and timely valve sizing is critical to achieving accurate measurements while the patient is euvolemic. Given that the RV and tricuspid valve geometry are volume-dependent, intraoperative sizing confirmation by transesophageal echocardiography could be very useful. Although we did not see chordal entrapment in the retrospective transesophageal echocardiography review, using an additional imaging modality, such as intracardiac echocardiography, could improve image quality and ensure adequate septal leaflet separation from the septum and appropriate valve placement. In addition, if there is any uncertainty regarding adequate capture of all leaflets, operators should abort the procedure before reaching a point at which corrective measures are no longer feasible. Furthermore, in the setting of suspicion for valvular embolization to the RV, surgical evaluation should be expedited immediately, as waiting could increase the risk of life-threatening complications. Finally, an ideal valve design should include an option for percutaneous valve retrieval or repositioning in the setting of early valve embolization.

## Conclusions

Although the TTVR has introduced remarkable improvements in quality of life and may provide a survival benefit in patients with severe tricuspid regurgitation, the current valve designs remain suboptimal. Valve migration, a potentially life-threatening complication, may reflect limitations of existing device design. Therefore, further improvement of current TTVR system is necessary to increase procedural safety and potentially improve clinical outcomes.

## Funding Support and Author Disclosures

The authors have reported that they have no relationships relevant to the contents of this paper to disclose.

**Visual Summary:** Embolization of Transcatheter Tricuspid Valve, From Diagnosis to Management

Initial presentation	Severe symptomatic tricuspid valve regurgitation despite optimal medical therapy and rhythm control.
Procedural planning	Coaptation gap of 15 mm, tricuspid valve annulus oversizing of 11.3% in the diastolic and 16.4% in the systolic phase, significant septal leaflet tethering.
Intraprocedural events	Valve deployment under meticulous echo guide. Slight valve shift after valve release leading to trace paravalvular leak.
Postprocedural events	Frequent arrythmia immediately after the procedure, and hemodynamic decompensation requiring extracorporeal membrane oxygenation cannulation and urgent surgery in postprocedure day 2.
Cardiac imaging	Transthoracic and transesophageal echocardiogram showed complete Evoque valve embolization to the right ventricle. Intraoperative imaging showed a large ventricular septal defect (VSD) created by the embolized valve.
Patient management	Surgical removal of Evoque valve, tricuspid valve replacement, and surgical VSD closure.
Follow-up	Patient recovered completely and was feeling well at 30-day follow-up.
